# Repeated examination of natural sapovirus infections in pig litters raised under experimental conditions

**DOI:** 10.1186/s13028-015-0146-7

**Published:** 2015-09-26

**Authors:** Klara Tølbøll Lauritsen, Mette Sif Hansen, Christina K. Johnsen, Gregers Jungersen, Blenda Böttiger

**Affiliations:** National Veterinary Institute, Technical University of Denmark, Bülowsvej 27, 1870 Frederiksberg C, Denmark; Department of Microbiological Diagnostics and Virology, Statens Serum Institut, Artillerivej 5, 2300 Copenhagen S, Denmark; TransMedEdit, Syvendehusvej 52, 2750 Ballerup, Denmark; Department of Laboratory Medicine, Medical Microbiology, Lund University, Sölvegatan 23, 22185 Lund, Sweden

**Keywords:** Sapovirus, Caliciviridae, Swine, RT-PCR, Phylogenetic analysis

## Abstract

**Background:**

Porcine sapovirus, belonging to the family *Caliciviridae*, is an enteric virus that is widespread in the swine industry worldwide. A total of 14 sapovirus genogroups have been suggested and the most commonly found genogroup in swine is genogroup III (*GIII*). The goal of the present experiment was to examine the presence of sapovirus in 51 naturally infected pigs at two different time points. The pigs were kept under experimental conditions after weaning. Previous studies on sapovirus have primarily been of a cross sectional nature, typically prevalence studies performed on farms and abattoirs. In the present study, faecal samples, collected from each pig at 5½ weeks and 15–18 weeks of age, were analysed for sapovirus by reverse transciptase polymerase chain reaction and positive findings were genotyped by sequencing.

**Results:**

At 5½ weeks of age, sapovirus was detected in the majority of the pigs. Sequencing revealed four different strains in the 5½ week olds—belonging to genogroups *GIII* and *GVII*. Ten to 13 weeks later, the virus was no longer detectable from stools of infected pigs. However, at this time point 13 pigs were infected with another *GIII* sapovirus strain not previously detected in the pigs studied. This *GIII* strain was only found in pigs that, in the initial samples, were virus-negative or positive for *GVII*.

**Conclusions:**

At 5 weeks of age 74 % of the pigs were infected with sapovirus. At 15–18 weeks of age all pigs had cleared their initial infection, but a new sapovirus GIII strain was detected in 25 % of the pigs. None of the pigs initially infected with the first *GIII* strain were reinfected with this new *GIII* strain, which may indicate the presence of a genogroup-specific immunity.

## Background

*Sapovirus* is a genus of the family *Caliciviridae*. Human sapovirus is known to cause acute gastroenteritis in children [1]. In swine, experimental infection with porcine sapovirus has also been associated with intestinal disease [2, 3]. In pig herd studies from several countries worldwide, sapoviruses have been reported to be present both in pigs with and without diarrhoea [4–12] leaving uncertainty about the magnitude of their role in enteric disease in pigs. Prevalence studies have shown that the virus is widespread and occurs worldwide in the pig industry, however, the reported prevalence varies from 3 to 67 % [5, 7, 10, 12–14]. Though the prevalence of sapovirus in pigs varies between countries, the highest prevalence is generally found in post-weaning pigs [7].

Sapoviruses are 35 nm sized non-enveloped viruses with a single-stranded positive-sense RNA genome of approximately 7.3 kb in length. The genome is composed of two open reading frames (*ORF*) where *ORF1* encodes the non-structural proteins and the major capsid protein, VP1, whereas *ORF2* encodes the minor structural protein VP2 [15, 16]. Based on the complete capsid gene sequences sapoviruses are divided into seven genogroups (*GI*-*GVII*) [15]. Pigs have frequently been reported to primarily host *GIII* sapoviruses [5, 8, 10, 12–14], but other studies have revealed a more extensive genetic heterogeneity among porcine sapoviruses. Thus a wider range of sapovirus genogroups including several proposed genogroups (*GVI* to *GXI*), have been detected in swine faeces [7, 9, 15, 17–21]. Based on the sequence of the RNA polymerase region, some of these swine sapovirus genogroups are more closely related to the human genogroups than to the *GIII* sapoviruses initially reported in swine.

The previous studies of sapoviruses in pigs have primarily been of a cross-sectional nature thus it is not known how sapovirus infections develop over time.

In the present study, we tested 51 post-weaning pigs twice for the presence of sapovirus. The pigs were naturally infected but kept under experimental conditions. Positive virus findings were characterized by sequencing, and the repeated sampling allowed comparison of the infections in each individual pig at two points in time.

## Methods

### Experimental set up

Two groups, A and B, comprised of 26 and 25 pigs respectively, were studied. The pigs were part of two identical vaccine trials against *Mycoplasma hyosynoviae*—a bacterium that causes arthritis and has no impact on the gastrointestinal system in infected swine. Therefore it was appropriate to conduct a parallel study of sapovirus occurrence in these animals. The two trials were run eight months apart in 2008 (A) and 2009 (B). The pigs were cross-breeds (Danish Landrace, Yorkshire, Duroc) and represented six age matched litters that originated from a Danish specific-pathogen-free swine herd. The herd was declared free from *Mycoplasma hyopneumoniae*, toxigenic *Pasteurella multocida*, *Bracyspira hyodysenteria*, Porcine reproductive and respiratory syndrome virus*, Haematopinus suis*, *Sarcoptes scabiei* and all *Actinobacillus pleuropneumoniae* serotypes except from serotype 12. Testing the herd for the presence of sapovirus was not performed prior to the experiment. The pigs were transferred to the experimental facilities after weaning at 3½–4 weeks of age and were kept there during the sampling period. As a standard procedure, the pens of the experimental facilities had been disinfected with 1 % Wofasteril 050 (BiOsense, Tønder, DK) (Group A) or 1 % Virkon S (Antec International, Sudbury, UK) (Group B) before use. In Group A three litters were distributed in four pens with 9 pigs (litter 1), 7 (litter 2), 5 and 5 pigs (litter 3) in each pen, respectively, without mixing the litters. In Group B three litters were distributed, without mixing the litters, in three pens of 9, 5 and 11 pigs, respectively. However, in Group B, at 7 weeks of age, a more even distribution of pigs in the three pens was desired and four pigs were transferred from the pen with the 11-pig litter to the pen with the smallest litter; thus obtaining 9, 9 and 7 pigs in the pens. Furthermore, in Group B, as part of a modification of the *M. hyosynoviae* infection model, at 13 weeks of age, half of the pigs in each pen were transferred to another of the three pens, thus mixing the litters within the second trial of the study.

The pens had concrete floors and bedding consisting of sawdust and straw. The pigs were fed a factory-made pelleted standard swine feed. No antimicrobials were added to the feed during the experiment. At weaning, all pigs had a normal body condition and did not show any clinical signs of disease. The study was approved by the Danish Animal Experiments Inspectorate (approval no. 2006/561-1106, protocol no. 70), and performed according to Danish legislation.

When the pigs were 5½ weeks old, faecal samples were collected manually from the rectum. Disposable plastic gloves were used for each sample and disposed between each sampling/pig. When the pigs were 15–16½ weeks old (Group A) or 16½–18 weeks old (Group B) follow-up faecal samples were obtained. All faecal samples were stored in sterile tubes at 4 °C until processing.

### Virus detection and sequencing

Faecal suspensions in 10 % phosphate buffered saline (PBS) were prepared, and after centrifuging RNA was extracted by using a total nucleic acids kit on a MagNa pure LC robot (Roche Diagnostics A/S, Hvidovre, Denmark). For the detection of sapovirus, two different reverse transciptase polymerase chain reaction (RT-PCR) tests, targeting the polymerase region, were used in all samples. In the first RT-PCR the primers used were p290/p289 [22] and in the second RT-PCR the same primers, in combination with four other p290/p289 derived primer pairs [23], were used. The PCR products were all 286 base pairs long exclusive of the primers. For RT-PCR, a one-step RT-PCR kit (QIAGEN Nordic, Copenhagen, Denmark) was used with the following reverse transcription and cycling conditions: a transcription step at 50 °C for 30 min, followed by 95 °C for 15 min and 40 cycles at 94 °C for 30 s, 49 °C for 30 s and 72 °C for 30 s with a final extension step at 72 °C for 10 min. The PCR products were sequenced in both directions on an ABI 3100 instrument using a BigDye kit v 1.1, (Applied Biosystems, Nærum, Denmark) and quality assessed and assembled using Bionumerics software (Applied Maths, Sint-Martens-Latem, Belgium). The sapovirus sequences obtained in the present study were deposited in GenBank with the following accession numbers: FJ947001, FJ947002, GU173811, GU173812 and GU320723.

The phylogenetic tree was constructed using the UPGMA clustering method (MEGA 4).

## Results

**Fig. 1:**
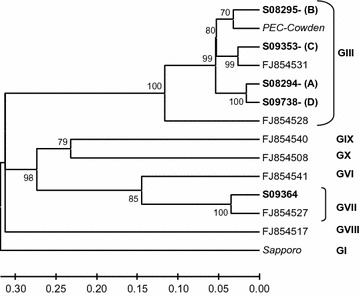
Phylogenetic tree based on 286 base pairs long RT-PCR sequences showing the sapovirus strains obtained. Five sapovirus strains were obtained in this study (shown in *bold*). The prototype strains PEC-Cowden (AF18276) and Sapporo (U65427) are shown in *italics*. The sequences designated with GenBank accession numbers are from a previous study [7]. The bootstrap values (500 replicates) are plotted at selected internal branch nodes. The tree is rooted with the out-group Sapporo (U65427). The *scale bar* represents nucleotide substitutions per site

The number of animals that tested positive for sapoviruses in relation to group, litter, sampling time, and genogroups is presented in Table 1. Sequencing of the PCR products showed that the sapoviruses belonged to two genogroups *GIII* and *GVII* (Fig. 1). Within *GIII*, four different strains, designated A–D, were detected. These strains differed 6–27 % in the nucleotide sequences and 4–10 % on amino acid level in the polymerase region (286 base pairs). However, all samples of the same strain (A: n = 15, B: n = 3, C: n = 5, D: n = 13) had 100 % identical nucleotide sequences in the polymerase region studied (286 base pairs).

**Table 1:** Detection of sapoviruses in faecal samples from naturally infected pigs

Pigs		Age 5½ weeks		Age 15–16½/16½–18 weeks	
	Litter no.	No. positive/no tested (%)	Sapovirus strain detected (no.)	No. positive/no. tested (%)	Sapovirus strain detected (no.)
Group A	1	9/9	GIII strain A (9)	0/9	
	2	2/5	GIII strain A (1) GIII strain B (1)	0/7	
	3	7/8	GIII strain A (5) GIII strain B (2)	0/10	
Total group A		18/22 (82 %)		0/26	
Group B	4	8/9	GIII strain C (5) GVII (3)	1/9	GIII strain D (1)
	5	1/5	GVII (1)	4/5	GIII strain D (4)
	6	8/11	GVII (8)	8/11	GIII strain D (8)
Total group B		17/25 (68 %)		13/25 (52 %)	
Total group A + B		35/47 (74 %)		13/51 (25 %)	

Detection of sapoviruses in faecal samples from pigs in relation to experimental group, litter, time of sampling and sapovirus genogroup (G) detected

GIII strain A: GenBank accession number FJ947001

GIII strain B: GenBank accession number FJ947002

GIII strain C: GenBank accession number GU173812

GIII strain D: GenBank accession number GU320723

GVII: GenBank accession number GU173811

In Group A, all follow-up samples were sapovirus negative, but in Group B, half of the pigs (n = 13) were sapovirus positive at the end of the experiment. Sequencing showed that these 13 pigs had been infected with a new sapovirus strain (*GIII* strain D) and that the sequences were 100 % identical. None of the two strains detected initially in Group B were found in follow-up samples. The *GIII* strain D was only detected in pigs that had been either sapovirus negative (n = 5) or *GVII* positive (n = 8) at 5½ weeks of age, and not in any of the five pigs that had been positive for *GIII* strain C at the first sampling.

## Discussion

Sapovirus infections are common in swine worldwide [7, 14, 18, 24]. The virus has been detected in pigs of all age groups, but is most frequently reported in post-weaning pigs [7, 11–14, 17, 24–26]. In the present study sapovirus was detected in 82 % of the pigs in Group A and 68 % of the pigs in Group B when sampled at 5½ weeks of age. The pigs originated from six different litters from one specific-pathogen-free herd, and they were healthy and in good condition at the initiation of the experiment.

The sapovirus strains detected in the pigs at 5½ weeks of age (*GIII* strain A, B, and C and *GVII*) could not be recovered in the second sampling when the pigs were 15–18 weeks old. However, we found that 13 of the 51 pigs excreted another sapovirus strain, *GIII* strain D, in the second sampling. Thus, in this experiment, more pigs were sapovirus-positive at 5½ weeks of age compared to 15–18 weeks. This indicates that pigs are transient shedders of sapovirus and confirms the findings of others [7, 14, 19] that sapovirus infections are highly prevalent in post-weaning pigs. However, Wang et al. [14] who investigated 621 faecal samples from American herds and abattoirs also found a high prevalence of sapovirus infections in finishers and sows, whereas they seemed to be less prevalent in nursing pigs. In Korea, Song et al. [19] investigated 567 faecal samples and found 37 sapovirus isolates of which 59.5 % derived from post-weaners, whereas 32.4 % were from nursing pigs and 8.1 % from growing pigs. It is possible that the more widespread detection of sapovirus across age-groups relates to a number of different sapovirus strains circulating within the farms and that older pigs will typically be sapovirus positive due to a reinfection. This was also observed in Group B in our study, where half of the pigs were sapovirus positive at 16½–18 weeks of age due to infection with a sapovirus of a new genogroup compared to earlier findings in the same pigs.

An interesting observation was that none of the pigs infected with a *GIII* virus at 5½ weeks of age, experienced any reinfection. The sapovirus strain causing reinfections in 13 pigs was also *GIII* (Table 1), but differed 7 % in the amino sequence in the polymerase region from the *GIII* strain causing the initial infection. This finding could indicate the existence of a genogroup-specific immunity, of at least a short duration. Furthermore, it also indicates the lack of cross-neutralization between the two different genogroups *GIII* and *GVII*. Such homotypic immunity has been described to be present in humans when infected with norovirus—another member of the *Caliciviridae* family [27, 28]. However, specific identification of a homotypic immunity in pigs infected with sapovirus and the duration of such immunity, would need further studies.

To improve the possibility of finding more than one genotype of sapovirus, two different RT-PCR tests, targeting the polymerase region, were used for the detection of sapoviruses in all samples. However, both test systems detected the same viruses in this setting. In another study, we detected different sapovirus genogroups and strains when testing faecal samples from swine with more than one RT-PCR assay. This was interpreted as double infections [7]. In the present study, five different sapovirus strains were detected, and the same virus strain was found in as many as 15 pigs. As sapoviruses are single-stranded RNA viruses with a high mutation rate, the finding of identical sequences indicates a common point source of infection. This source could be the housing environment or other animals e.g. the sow. Strict isolation rules were applied in the stable with respect to outer surroundings, but not between the pens within the experiment. Therefore spread of infection from animals in one pen to another may have occurred by vehicle transmission by e.g. footwear, personnel or tools. For unknown reasons we found an additional strain (*GIII* strain D), not earlier detected in this study, in the second sampling in Group B. This was unexpected since we would expect that all sapovirus sequences in the study would have originated from the herd of origin. However, sapoviruses can survive outside the host for considerable time and thorough disinfection with special disinfectants is needed to inactivate the virus [15]. The litters in Group B were mixed twice before the second sampling, a fact that might have affected the results as the pigs could have been in contact with new surroundings and viruses during the transfers. The introduction of the *GIII* strain D might be explained by either, insufficient disinfection, introduction via the personnel or introduction via bedding material/feed. The reason still remains unidentified.

## Conclusions

We found that the vast majority of the 5½ week old pigs carried sapovirus whereas the infection, though present, was less frequent in the pigs in the second sampling at 15–18 weeks of age. Pigs shedding sapovirus at the second sampling all had an infection with a different genotype than at the first sampling, indicating that they had cleared the initial infection. Thus, reinfection occurs, but genogroup-specific immunity might exist for sapovirus, at least of a short duration, such as it has been described for other members of the *Caliciviridae* family in humans. If this is the case, it would be relevant to investigate the duration of such immunity and in addition, which parts of the immune system may be providing such immunity.

## Abbreviations

*GIII*: sapovirus genogroup III
*GVI*: sapovirus genogroup GVI
*GVII*: sapovirus genogroup VII
*GXI*: sapovirus genogroup GXI
ORF: open reading frame
PBS: phosphate buffered saline
PCR: polymerase chain reaction
RNA: ribonucleic acid
RT-PCR: reverse transciptase polymerase chain reaction
VP1: major capsid protein of sapovirus
VP2: minor structural protein of sapovirus
